# Structural Basis for The Recognition of Deaminated Nucleobases by An Archaeal DNA Polymerase

**DOI:** 10.1002/cbic.202100306

**Published:** 2021-09-14

**Authors:** Heike M. Kropp, Samra Ludmann, Kay Diederichs, Karin Betz, Andreas Marx

**Affiliations:** ^1^ Department of Chemistry University of Konstanz Universitätsstraße 10 78457 Konstanz Germany; ^2^ Department of Biology University of Konstanz Universitätsstraße 10 78457 Konstanz Germany; ^3^ Konstanz Research School Chemical Biology University of Konstanz Universitätsstraße 10 78457 Konstanz Germany

**Keywords:** crystal structure, deaminated base recognition, DNA polymerases, hypoxanthine, uracil

## Abstract

With increasing temperature, nucleobases in DNA become increasingly damaged by hydrolysis of exocyclic amines. The most prominent damage includes the conversion of cytosine to uracil and adenine to hypoxanthine. These damages are mutagenic and put the integrity of the genome at risk if not repaired appropriately. Several archaea live at elevated temperatures and thus, are exposed to a higher risk of deamination. Earlier studies have shown that DNA polymerases of archaea have the property of sensing deaminated nucleobases in the DNA template and thereby stalling the DNA synthesis during DNA replication providing another layer of DNA damage recognition and repair. However, the structural basis of uracil and hypoxanthine sensing by archaeal B‐family DNA polymerases is sparse. Here we report on three new crystal structures of the archaeal B‐family DNA polymerase from *Thermococcus kodakarensis* (KOD) DNA polymerase in complex with primer and template strands that have extended single stranded DNA template 5’‐overhangs. These overhangs contain either the canonical nucleobases as well as uracil or hypoxanthine, respectively, and provide unprecedented structural insights into their recognition by archaeal B‐family DNA polymerases.

## Graphical abstract

**Due to their life** in hot environments, archaea are increasingly affected by deamination of the bases cytosine and adenine. To prevent mutations, archaeal DNA polymerases seem to recognize these base changes by a so‐called “read‐ahead” mechanism and react accordingly. Here we show by means of crystal structures how *Thermococcus kodakarensis* (KOD) DNA polymerase binds uracil and hypoxanthine in a special binding pocket and thus can recognize these bases on the single stranded template overhang already six nucleotides before the active site.

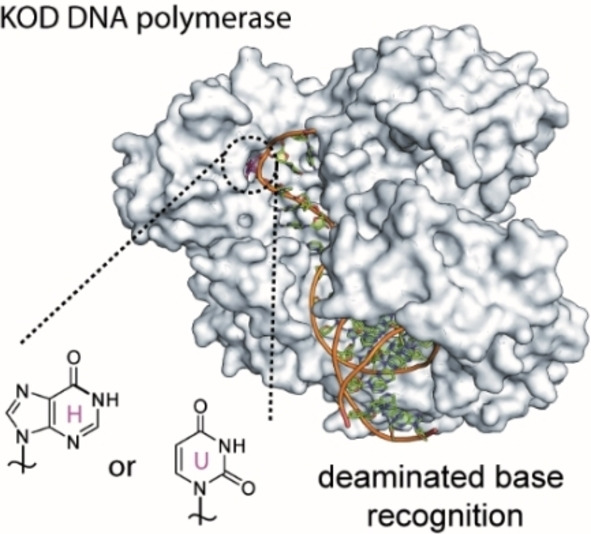

## Introduction

**Figure 1:**
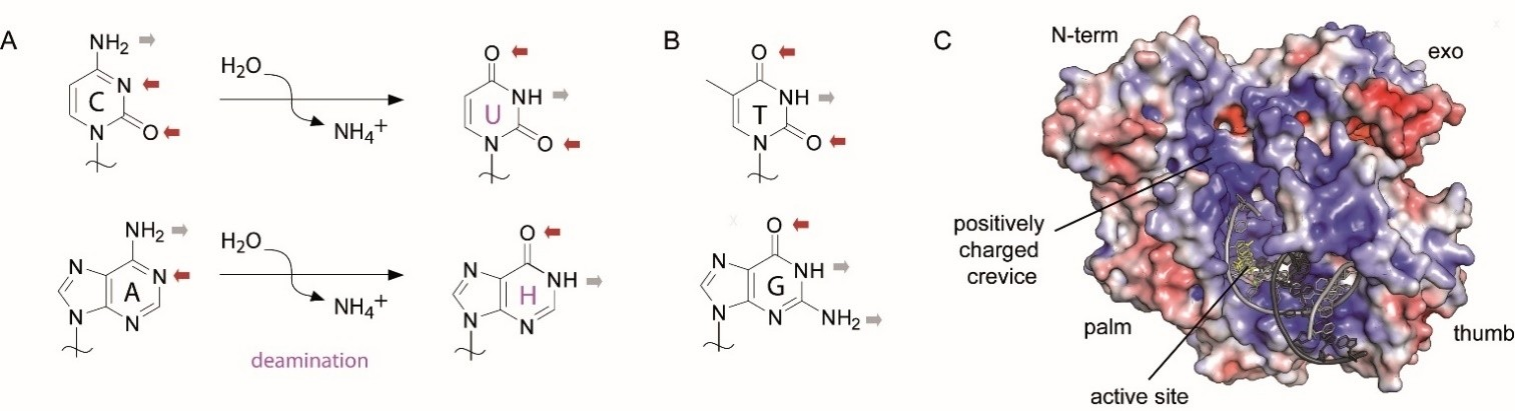
A) Deamination of cytosine to uracil and adenosine to hypoxanthine leads to a change in hydrogen bonding donor and acceptor pattern. Grey arrows show hydrogen bonding donors, red arrows show hydrogen bonding acceptors. B) Thymidine and guanosine seem to be sterically excluded from the uracil/hypoxanthine recognition pocket due to their 5‐CH_3_ and 2‐NH_2_ groups, respectively. C) Electropotential map of KOD DNA polymerase (PDB ID: 4K8Z). The electropotential is shown from +5 (blue) to −5 (red) kBT/e (T=310 K). Primer and template are shown as dark and light grey cartoon, respectively and the substrate dATP in the active site is shown as yellow sticks.

Several archaea live at temperatures exceeding 80 °C and since DNA is prone to deamination at elevated temperatures, this results in increased cytosine to uracil and adenine to hypoxanthine conversions (Figure 1 A).^[1]^ These alterations will lead to G : C to A : T and *vice versa* transition mutations in the genome and therefore, their genomic integrity is at higher risk.^[1]^ All organisms contain repair systems^[2]^ that specifically recognize and excise damaged nucleotides e. g., uracil from DNA by glycosidic bond cleavage that initiates the base excision repair pathway^[3]^ and restores the G : Cbase pair. Interestingly, Connolly and colleagues found that archaeal B‐family DNA polymerases have evolved a so called “read ahead” mechanism to sense uracil in the template strand, stalling DNA polymerase promoted DNA synthesis of nascent DNA before the deaminated nucleobase reaches the polymerase active site.^[4]^ Similar results were also obtained when hypoxanthine was studied as deaminated nucleobase in the template strand.^[5]^ Noteworthy, the predicted uracil and hypoxanthine binding pocket is highly conserved among archaeal B‐family DNA polymerases.[^4b^, ^4e^] A reliable detection of uracil and hypoxanthine besides the four natural bases (structural differences see Figure 1A and B) poses great challenges for the binding pocket, which are met through specific interactions and steric aspects.^[4f]^ First structural models into the process of uracil recognition by archaeal B‐family DNA polymerases were obtained from a *Thermococcus gorgonarius* (Tgo) DNA polymerase in complex with a DNA hairpin structure, that mimics a primer/template (p/t) complex containing a uracil at the +4 position (+1 is the first single stranded nucleotide) in the single stranded template region (PDB ID: 2VWJ, termed Tgo‐U here).^[4c]^ The hairpin is bound in a way that uracil is located in the pocket and the primer end is placed somewhere between the polymerization active site and the exonuclease active site (Figure S4A; for details see ref. [4c]). A similar Tgo DNA polymerase structure with a DNA construct containing hypoxanthine at position +2 (two bases ahead of the p/t junction, PDB ID: 2XHB, termed Tgo‐H here)^[4f]^ shows that hypoxanthine can be recognized via binding in the same pocket as uracil. In comparison to the structure Tgo‐U, in which all primer/template bases in the stem region of the hairpin are paired, the last two primer nucleotides are single stranded and point into the editing cleft (see black nucleotides in Figure S4B). However, the primer end does not reach the active site of the exonuclease domain and it is presumed that further conformational changes are necessary to reach it (for more details on stimulation of the 3’‐5’ exonuclease proofreading activity of Tgo upon encountering a deaminated base in the template see ref. [4f]). Structures of B‐family DNA polymerases with templates containing deaminated bases and the p/t complex bound in a ‘productive’ complex are missing.

In general, template recognition by archaeal B‐family DNA polymerases is poorly understood on the structural level due to the lack of structural data of the respective DNA polymerases containing template, primer and dNTP with longer single stranded 5’‐template overhangs despite the fact that they are extensively used for biotechnological applications such as PCR and DNA sequencing.^[6]^ Structures that were recently published by us identified a positively charged crevice between the N‐terminal and exonuclease domain (Figure 1C) thought for template 5’‐overhang binding, but the used template 5’‐overhangs were too short, and little resolved to draw further structural conclusions.^[7]^ Overall, our structural understanding of uracil and hypoxanthine sensing is poor.

Here we report on three new crystal structures of the archaeal B‐family DNA polymerase from *Thermococcus kodakarensis* (KOD) DNA polymerase in complex with extended single stranded 5’‐overhangs. These overhangs contain either the canonical nucleobases as well as uracil or hypoxanthine, respectively, and provide unprecedented structural insights into their recognition by archaeal B‐family DNA polymerases.

## Results and Discussion

### Structure of KOD DNA polymerase with an extended single stranded template 5’‐overhang

**Figure 2:**
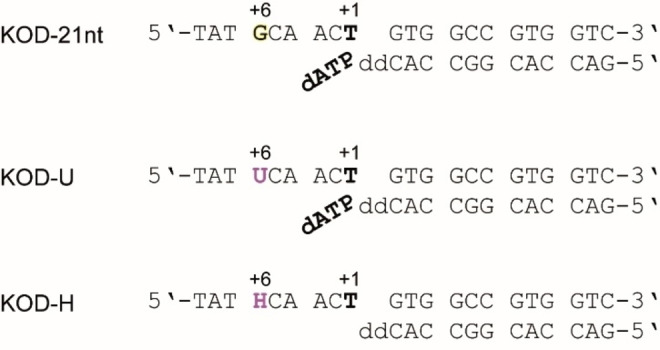
The template (top) and primer (bottom) sequences used for the crystallization of KOD‐21nt, KOD‐U and KOD‐H. The nucleotide incorporation site is indicated in bold and assigned position +1. For the ternary complexes KOD‐21nt and KOD‐U the bound dATP is shown in bold.

In a previously solved ternary KOD DNA polymerase structure (termed KOD‐dATP here, PDB ID: 5OMF)^[7b]^ an electropositive crevice between the N‐terminal and exonuclease domain was observed (Figure 1C) which is suspected to be the binding site of the single stranded template overhang. As the template overhang in the earlier structure was short and not well resolved, we aimed to investigate the exact binding mode of the template using a longer template. Therefore, we set up co‐crystallization trials employing a 21 nucleotide (nt) template (Figure 2). Much to our delight, the 21 nt template showed enhanced crystallization behavior of KOD DNA polymerase, compared to the 16 nt template used before^[7b]^ and data from various crystallization conditions could be obtained.

**Figure 3:**
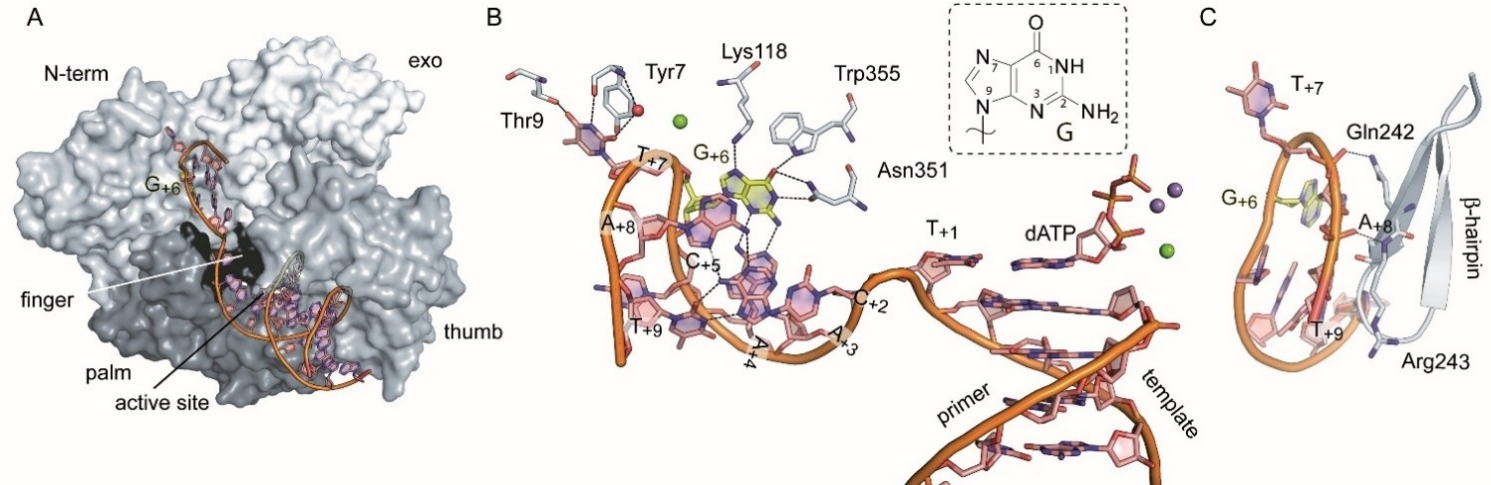
KOD DNA polymerase in complex with a 21 nt template. A) The protein surface is shown and the domains are in different shades of grey and labelled. The backbone of the primer/template (p/t) complex is shown in orange. The single stranded template overhang is located in the crevice between the N‐terminal and the exonuclease domain B) Detailed view on the p/t complex. The thymidine at position +7 (T_+7_) forms hydrogen bonds to amino acids of the N‐terminal domain as well as π‐π stacking interactions to Tyr7. Non‐canonical base pairing between nucleobases in the single stranded template region is indicated by black dashed lines. The substrate dATP is shown as sticks and the metal ions Mg^2+^ and Mn^2+^ in the active site are shown as green and violet spheres, respectively. (C) The β‐hairpin is located alongside the single stranded template and forms hydrogen bonds to the phosphate backbone.

The structure of KOD DNA polymerase with the 21 nt template (henceforth termed KOD‐21nt) and the 12 nt primer, used before, is in a closed ternary complex, with dATP and three metal ions (Mg^2+^: A‐site, Mn^2+^: B‐ and C‐site) bound in the active site (Figure 3A, B). Overall, the structure resembles the earlier published closed structure KOD‐dATP (rmsd value 0.180 for 700 Cα atoms) and shows the same metal ions and triphosphate coordination in the active site. In the structure all nucleotide residues could be modelled in the electron density map. The single stranded template moves along the N‐terminal domain and folds as a loop structure into the positively charged crevice between N‐terminal and exonuclease domain (Figure 3A). The last two nucleotides are arranged, parallel to a β‐hairpin that was shown to play a role in stabilizing the melted DNA when bound in the exonuclease active site.^[8]^ Here, the β‐hairpin (specifically the side chain of Gln242 and the backbone of Arg243) forms two hydrogen bonds to the phosphate backbone of T_+7_ and A_+8_ (Figure 3C). T_+7_ forms further hydrogen bonds to the amino acids Thr9 and Tyr7 of the N‐terminal domain as well as a π‐π stacking interaction with the amino acid side chain of Tyr7 (Figure 3B) and a cation‐π crystal contact to K429 of the next molecule of the crystal lattice (data not shown), that seems to stabilize the template in the crevice. The nucleobases A_+4_, G_+6_ and T_+9_ as well as A_+3_ and A_+8_ build non‐canonical base pairs (hydrogen bonds depicted in Figure 3B), that may stabilize the template in this looped conformation.

Superposing KOD‐21nt with the previously solved structure of Tgo DNA Polymerase with an uracil‐containing template (Tgo‐U), we identified that the binding pocket responsible for the recognition of deaminated nucleobases (further termed uracil/hypoxanthine binding pocket) described in the introduction is in close proximity to the +6 position of the single stranded template overhang when the enzyme is in an active polymerization state. Here, the binding pocket is occupied by an ethylene glycol molecule that was present in the crystallization condition (Figure S3A, B). The guanosine located at position +6 is sterically excluded from this pocket (see next section). Instead, it points into a different direction where it is surrounded by template residue A_+4_, A_+8_ and the amino acids Pro115, Lys118, Asn351, Asp343, Trp355 and Lys371 (Figure S1C, D). The guanine nucleobase seems very well stabilized at this position engaging specific hydrogen bonds via its 1‐NH, 2‐NH_2_, N3 O6 and N7 atoms to Lys118, Trp355 and Asn351 as well as to A_+4_ (Figure 3B and S1C, D). We speculate that also the other natural nucleotides could bind at this position and engage similar interactions.

### Structure of KOD DNA polymerase with an extended single stranded template 5’‐overhang containing a uracil

**Figure 4:**
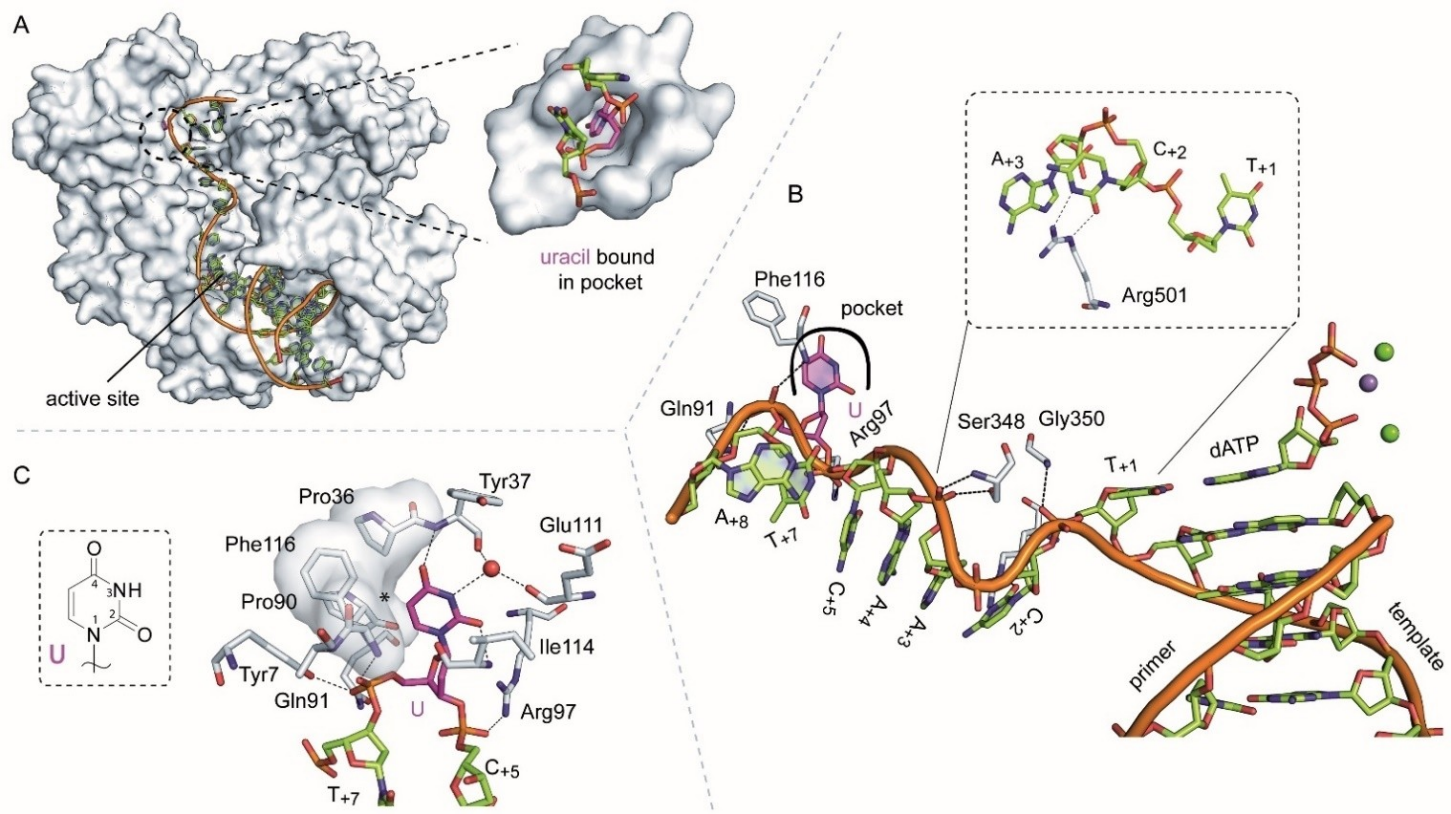
A) Overall structure of KOD‐U shown as surface and the p/t duplex shown as cartoon. The single stranded template overhang binds into the crevice between the N‐terminal and the exonuclease domain. A zoom into the pocket in which uracil binds in the N‐terminal domain is shown below. B) Detailed view on the single stranded part of the p/t complex (standard nucleotides in green and uracil in pink). Protein residues interacting with the single‐stranded part are shown in grey. C) Residues forming the pocket in which uracil binds are shown as sticks. Interactions of uracil and flanking phosphate moieties are shown. The asterisk indicates the position where the methyl group of a bound thymidine could cause steric clashes. The chemical structure of uracil is shown in the box.

To verify if a uracil nucleobase at position +6 would bind into the uracil/hypoxanthine binding pocket we performed crystallization experiments with the uracil‐containing 21 nt template and KOD DNA polymerase and obtained a closed ternary complex structure just as for KOD‐21nt (henceforth termed KOD‐U, Figure 4A). The overall structures of KOD‐U and KOD‐21nt are very similar (rmsd value 0.386 Å, for 697 Cα atoms). In the active site three metal ions (Mg^2+^: A‐ and C‐site, Mn^2+^: B‐site) are coordinated by the substrate dATP and the residues Glu580, Asp404 and Asp542 characteristic for a complex trapped prior to catalysis^[7b]^ (Figure 4B). The single stranded template overhang could be traced in the electron density map up to the nucleotide A_+8_ while the last nucleotide T_+9_ was too flexible to be modelled. The single stranded DNA points into the already described electropositive crevice between the N‐terminal and exonuclease domain, however the exact conformation of the nucleotides is quite different compared to KOD‐21nt. Instead of forming a loop structure the template is fully extended with all nucleobases (except the uracil) pointing towards the solvent (compare Figure 3B and Figure 4B). The kink of the template backbone between T_+1_ (which base pairs with the substrate dATP) and the first single stranded template nucleotide C_+2_ is more pronounced. In detail, the Watson‐Crick side of C_+2_ points to the complete opposite direction and its position seems to be stabilized by hydrogen bonds to Arg501 and additional stacking arrangement of Arg501 and C_+2_ together with A_+3_. (Figure 4B, zoom in). The nucleobases of A_+4_ and C_+5_ continue the stacking arrangement on A_+3_. In addition to electrostatic effects several hydrogen bonds between the protein and the phosphate backbone can stabilize the extended template conformation. The following residues are involved: Tyr7, Gln91, Phe116 (with U_+6_), Arg97 (with C_+5_) Ser348 (with A_+3_), Gly350 (with T_+1_).

The uracil nucleobase at position +6 of the template points away from its flanking bases and is accommodated in a pocket formed by the amino acids Tyr7, Glu35, Pro36, Tyr37, Pro90, Gln91, Val93, Pro 94, Arg97, Glu111, Tyr112, Asp113, Ile114, Pro115, Phe116, Ala117 and Arg119 (Figure 4A and S3B; all residues containing atoms surrounding uracil in a radius of 5 Å were selected). This pocket was previously described in detail in the work of Connolly and colleagues using Tgo DNA polymerase.^[4c]^ Both, the structure of the pocket found in our KOD‐U structure and the earlier Tgo DNA polymerase derived structure Tgo‐U (PDB ID: 2VWJ) superimpose very well (see Figure S4A, C) and the uracil residues are snugly bound there in *anti*‐conformation in both structures. The amino acids forming the pocket are highly conserved in archaeal B‐family polymerases (only one amino acid that is at the back of the pocket differs between Tgo and KOD DNA polymerases: Phe38 in KOD is Ile38 in Tgo). The phosphates immediately 5’ and 3’ to the uracil show interactions with the side chains of Gln91, Tyr7 and Arg97 as well as the backbone of Phe116 (as mentioned above). Identical to the interactions observed by Connolly and colleagues, inside the pocket, uracil shows hydrogen bonds from O2 and O4 to the backbone amide nitrogen atoms of Ile114 and Tyr37, respectively (Figure 4C and S3C). In KOD‐U, an additional hydrogen bond is formed between the 3‐NH group and a water molecule which itself interacts with the backbone carbonyl groups of Tyr37 and Glu11 as well as the side chain of Arg119 (not shown in Figure 4B).

All of these interactions, together with the steric aspects of the binding pocket, result in the uracil being perfectly bound. This can also be seen in the lower flexibility, i. e. the lower B‐factors of uracil, compared to the neighboring nucleotides (Figure S5). A key feature of uracil versus thymine discrimination is the space available at the position of the C5‐methyl group. At this position the edge of the pocket is formed by Pro36, Pro90 and Phe116 (Figure 4C, marked by an asterisk). If thymine is superimposed on the uracil base, distances of 2.8 to 3 Å to the neighboring residues are measured for the methyl group, which can lead to clashes that excludes thymidine to bind into the pocket as also concluded in earlier studies by Connolly and colleagues.^[4]^ While thymine is sterically excluded, stable binding of cytosine into the pocket can be excluded due to its different hydrogen bonding donor and acceptor pattern (Figure 1A, B). As mentioned above KOD‐U was trapped in an active polymerization complex with a substrate dATP bound. This is in contrast to the Tgo‐U structure reported earlier. Here the DNA hairpin is bound such that the duplex end is situated somewhere between the polymerization and exonuclease active site (Figure S4A). Therefore, our structure might show the first recognition step of uracil during DNA replication six nucleotides ahead of the active site, while the Tgo‐U structure could represent an intermediate step in the transition to binding of the primer end in the exonuclease active site.

### Structure of KOD DNA polymerase with an extended single stranded template 5’‐overhang containing a hypoxanthine

**Figure 5:**
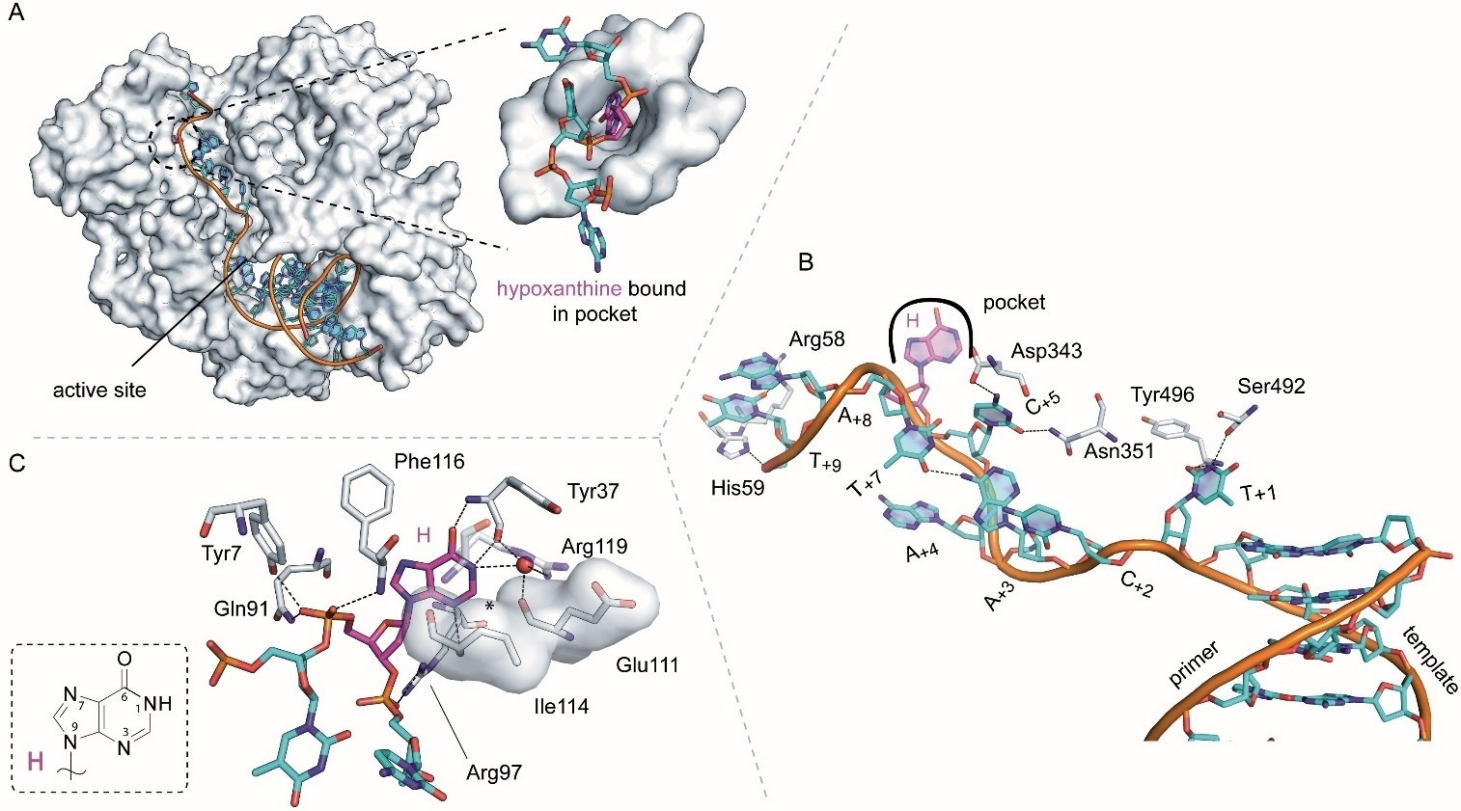
A) Overall structure of KOD‐H shown as surface and the primer/template (p/t) duplex shown as cartoon. The single stranded template overhang binds into the crevice between the N‐terminal and the exonuclease domain. Hypoxanthine (shown in pink) points into a pocket in the N‐terminal domain. A zoom into the pocket is shown. B) Detailed view on the single stranded part of the p/t complex with standard nucleotides shown in cyan and hypoxanthine shown in pink. Protein residues interacting with the single‐stranded part are shown in grey. C) Interactions of hypoxanthine and flanking phosphate moieties with the protein. The asterisk indicates the position where the amino group of a bound guanine could cause steric clashes. The chemical structure of hypoxanthine is shown in the box.

Besides uracil it was shown that also hypoxanthine can be recognized in the template strand before reaching the active site by binding of hypoxanthine into the uracil/hypoxanthine binding pocket.^[4f]^ To study this in our context we performed analogous crystallization experiments as described above with a hypoxanthine‐containing 21 nt template (Figure 2). The experiments resulted in a binary complex with the p/t complex bound by the enzyme in an open state, characterized by an open finger domain and no substrate triphosphate bound. The structure is henceforth termed KOD‐H. The overall structure superposes well with the previously published binary KOD DNA polymerase structure with a 16 nt template (PDB ID 4K8Z,^[7a]^ rmsd 0.355 Å, for 702 Cα atoms). The single stranded template overhang is positioned in the same cleft as in KOD‐21nt and KOD‐U and the hypoxanthine at position +6 binds in the same pocket as the uracil nucleotide in KOD‐U (Figure 5A, overlay see Figure S3B). All single stranded nucleotides could be modelled, however, the single stranded nucleotides C_+2_, A_+3_ and A_+4_ show weaker electron density and hence more flexibility compared to the rest of the template and compared to the same positions in KOD‐U. These nucleotides may adopt different alternate conformations. The first single stranded nucleotide T_+1_ does not stack on the p/t duplex but is rotated to the side and the nucleobase is twisted by nearly 90° compared to the p/t duplex end (Figure 5B). At this position the nucleobase of T_+1_ can be well stabilized forming hydrogen bonds to the backbone oxygen of Ser492 and the backbone nitrogen of Tyr496. In the previously solved binary KOD structure with a shorter and fully natural single stranded template overhang (PDB ID: 4K8Z^[7a]^) the first single stranded nucleotide was found in a slightly different position and showed more flexibility with no obvious stabilizing interactions. As mentioned above, C_+2_, A_+3_ and A_+4_ are quite flexible and show no specific interactions with the enzyme. C_+5_ is pointing towards the enzyme and can engage hydrogen bonds with Asn351, Asp343 and Trp355 (Figure 5B). Hypoxanthine is bound in the same pocket as uracil and all residues involved superpose well with the ones in KOD‐U (Figure S3B) as well as with the pocket in the previously solved Tgo‐H (PDB ID: 2XHB) structure (Figure S4D). Again the backbone amide nitrogen atoms of Tyr37 and Ile114 interact with the exocyclic O6 and the ring N3 of hypoxanthine, respectively while a water molecule, that is bridged by hydrogen bonds to Glu111, Tyr37 and Arg119 interacts with the 1‐NH group (Figure 5C and S3C). The B‐factors of hypoxanthine are lower compared to the neighboring nucleotides which speaks for a stabilization in the binding pocket (Figure S5). The last nucleotide T_+9_ seems well stabilized forming hydrogen bonds with Arg58 and His59 and stacking between the histidine side chain and the residue A_+8_ (Figure 5B).

The side of the pocket where the exocyclic amine group of guanine would be placed is bordered by Glu111 and Ile114 (Figure 5C, marked by asterisk). Superimposition of guanine on hypoxanthine reveals a clash of 2‐NH_2_ with the main chain oxygen of Glu111 (nearest distance around 2.2 Å) or the side chain of Ile114 (nearest distance around 2.7 Å) explaining guanine exclusion. Adenosine can be excluded from binding into the pocket due to its reverse hydrogen bonding pattern compared to hypoxanthine (donor‐acceptor for adenosine versus acceptor‐donor for hypoxanthine) that does not allow the described stabilizing hydrogen bonds (see Figure 1).

It is surprising that despite the difference in size, both uracil and hypoxanthine bind selectively in the same binding pocket. An overlay of the protein residues forming the pocket in KOD‐U and KOD‐H (Figure S3B) shows that they are positioned almost identically. The only significant difference is a water molecule which participates in hydrogen bonds to 3‐NH of uracil and 1‐NH of hypoxanthine. In KOD‐H, the water molecule is displaced by 0.9 Å to accommodate the bigger nucleobase. The water molecule can still be stabilized by the same residues (namely Tyr37, Glu111, Arg119) but the distance to Arg119 decreases from 3.3 to 2.9 Å.

## Conclusion

Here we report the first crystal structures of an archaeal B‐family DNA polymerase that reveal the positioning of the single stranded DNA template overhang within the DNA polymerase complex. The template overhang binds into a positively charged crevice between the N‐terminal and the exonuclease domain of the DNA polymerase. Most interestingly we identified that the pocket that was previously shown to be involved in a read ahead sensing of deaminated nucleobases (uracil/hypoxanthine pocket) is located in close proximity to the template residue G at position +6 in KOD‐21nt. To further investigate this we solved analogous crystal structures with uracil or hypoxanthine at position +6 and indeed both nucleobases fit snugly into the pocket and show various interactions with the enzyme. The nucleobases also show lower flexibility compared to their neighboring residues which supports the tight binding. The pocket in which uracil and hypoxanthine bind is also present in KOD‐21nt and all residues involved superpose well in the three structures. This indicates that the pocket does not adapt to the nucleotide structure upon binding but is preformed to recognize the deaminated bases. While guanine and thymine are sterically excluded from the pocket due to clashes with their exocyclic 5‐CH_3_ or 2‐NH_2_ groups. Adenine and cytosine can be excluded due to their differing hydrogen bonding donor and acceptor patterns.

Previous structures of Tgo DNA Polymerase in complex with uracil‐ or hypoxanthine‐containing templates were trapped with uracil at template position +4 or hypoxanthine at template position +2. In both structures the enzyme is not in a polymerization state but rather shifted towards an editing mode. The herein reported structures suggest that during replication deaminated nucleobases might already be recognized six nucleotides ahead of the active site. If polymerization then continues the deaminated nucleobases bind to their pocket hindering correct translocation and introducing tension in the p/t complex that could stall replication or activate exonuclease activity as discussed earlier.

Taking together, we herewith report on three new structures that provide structural insights into how archaeal B‐family DNA polymerases are recognizing the single stranded DNA template overhang in the presence of canonical and deaminated nucleobases. Since these enzymes are extensively used in biotechnological applications these insights might guide for engineering endeavors in order to improve DNA polymerase properties for advanced applications.

## Experimental Section

### Expression and purification of KOD DNA polymerase

For the crystallization experiments the exonuclease deficient mutant D141A, E143A was used. KOD DNA polymerase was cloned into a pET24a vector (5′: NdeI, 3′: NotI) and overexpressed in *E. coli* BL21 (DE3) using a codon‐optimized sequence as described by Bergen et al.^[7a]^ LB‐media (2 L) containing 34 mg/L kanamycine were inoculated with overnight culture to a final OD_600_=0.05–0.1. The cells were grown at 37 °C to an OD_600_ of 0.6, followed by cooling the media on ice to approx. 15–20 °C and inducing the expression using 1 mM IPTG. The cells were further grown at 15–20 °C, 190–200 rpm overnight and harvested at 4000 rpm. Further purification steps were done as described by Kropp et al.^[7b]^

### Crystallization conditions

dATP and ddCTP were purchased from Jena Bioscience and stored at −20 °C as 100 mM or 10 mM solutions. Oligonucleotides were purchased HPLC purified from MWG Eurofins and Biomers and were used without further purification. Primers and templates were stored in “oligo‐annealing buffer” (10 mM Tris pH 7.5, 50 mM NaCl, 1 mM EDTA) as 6 mM solutions. Primer and template were mixed in a ratio of 1 : 1 and were heated to 95 °C for 5 min, followed by the stepwise cooling (2 °C per 20 sec) to 4 °C. For crystallization the purified enzyme was mixed with the respective p/t duplex, and ddCTP in a 1 : 1.2 : 3 molar ratio and the solution was incubated at 55 °C for 30 min. Afterwards, dATP was added in a 10 times molar excess compared to the enzyme (final concentration of dATP: 1.36 mM) and the solution was incubated at 30 °C for 45 min. Prior to crystallization the solutions were filtered through a 0.1 μm centrifugal filter (Ultrafree®‐MC‐VV, Durapore® PVDF, Millipore) at 4000 rpm and stored at room temperature.

### KOD‐21 nt

Crystals grew in the condition E7 of the Morpheus screen (Molecular Dimensions) containing 0.12 M ethylene glycols (0.3 M diethylene glycol, 0.3 M triethylene glycol, 0.3 M tetraethylene glycol, 0.3 M pentaethylene glycol), 0.1 M sodium HEPES/MOPS pH 7.5, 30 % (v/v) of a mixture of glycerol (40 % v/v) and PEG 4000 (20 % w/v). KOD‐21nt crystals could also be reproduced in the G3, F7, H7, D7 and F3 conditions of Morpheus® screen. The final protein concentration (after mixing with p/t complex) used for the setups was 8.3 mg/ml.

### KOD‐U

Crystals grew in condition A3 of the Morpheus screen containing 0.06 M divalents (0.3 M magnesium chloride hexahydrate; 0.3 M calcium chloride dihydrate) 0.1 M buffer system 1 (1 M MES/imidazol pH 6.5) 30 % v/v precipitant mix 3 (40 % v/v Glycerol; 20 % w/v PEG 4000). The final protein concentration (after mixing with p/t complex) used for the setups was 8.3 mg/ml.

### KOD‐H

Crystals grew in the condition B12 of the Morpheus screen containing 0.09 M halogens (0.3 M sodium fluoride; 0.3 M sodium bromide; 0.3 M sodium iodide) 0.1 M buffer system 3 (1 M Tris (base); BICIN, pH 8.5) 37.5 % v/v precipitant mix 4 (25 % v/v MPD; 25 % PEG 1000; 25 % w/v PEG 3350). The final protein concentration (after mixing with p/t complex) used for the setups was 6.2 mg/ml.

All crystals were cryo‐protected with 20 % ethylene glycol in the reservoir solution before flash freezing in liquid nitrogen.

### Data collection

Data was collected at the Swiss Light Source (SLS) of the Paul Scherrer Institute (PSI) in Villigen, Switzerland at the beamline PXI X06SA – Eiger 16 M detector. Data collection and refinement statistics are summarized in Table 1 in the Supporting Information.

### Structure determination

Data were processed using the XDS package^[9]^ and the XDSGUI graphical interface (https://strucbio.biologie.uni‐konstanz.de/xdswiki/index.php/XDSGUI). The structures were solved by rigid body refinement against published KOD DNA polymerase structures of the same spacegroup (PDB: 5OMF or 4K8Z). Structure refinement was done with phenix.^[10]^ Model building was done in Coot^[11]^ and model quality was evaluated by the MolProbity^[12]^ web server (http://molprobity.biochem.duke.edu) and the PDB validation server (https://validate‐rcsb‐1.wwpdb.org). Binding of Mn^2+^ ions in the active site of the enzyme or at other locations of the protein was determined by anomalous signal. Therefore, Bijvoet pairs were use separately in refinement, and the anomalous signal was accounted for in the model. The occupancy of metal ions and ligands was refined if difference density appeared during refinement. Figures were created with PyMOL.^[13]^ For superimposition in figures and rmsd value calculation, structures were superimposed in PyMOL using the “align” command. Electropotential maps were generated with the APBS plugin for PyMOL (see https://pymolwiki.org/index.php/APBS). Final refined 2mFo‐DFc electron density maps as well as polder maps^[14]^ are shown for the residues G_+6_, uracil and hypoxanthine in Figure S1A, B and S2.

## Conflict of interest

The authors declare no conflict of interest.

## Supporting information

As a service to our authors and readers, this journal provides supporting information supplied by the authors. Such materials are peer reviewed and may be re‐organized for online delivery, but are not copy‐edited or typeset. Technical support issues arising from supporting information (other than missing files) should be addressed to the authors.

Supporting Information
