# Supplementary material for: Structural Basis for The Recognition of Deaminated Nucleobases by An Archaeal DNA Polymerase
Source: Chembiochem. 2021 Sep 14;22(21):3060–6. doi: 10.1002/cbic.202100306 (PMC8596578; doi:10.1002/cbic.202100306)
Supplement: Supplementary file 1 — Supporting Information [file CBIC-22-3060-s001.pdf]

ChemBioChem

Supporting Information

Structural Basis for The Recognition of Deaminated Nucleobases by An Archaeal DNA Polymerase

Heike M. Kropp, Samra Ludmann, Kay Diederichs, Karin Betz,* and Andreas Marx*

Structural basis for the recognition of deaminated nucleobases by an archaeal DNA polymerase

	**KOD-21nt**	**KOD-U**	**KOD-H**
**PDB ID**	**7OMB**	**7OMG**	**7OM3**
Wavelength (Å)	1.0	1.0	1.0
Space group	$P2_12_12$ (18)	$P2_12_12$ (18)	$P2_12_12$ (18)
**Cell dimensions**			
a, b, c (Å)	108.08, 147.15, 71.33	108.38, 147.08, 71.10	113.26, 142.94, 65.58
α, β, γ (°)	90.00, 90.00, 90.00	90.00, 90.00, 90.00	90.00, 90.00, 90.00
Resolution (Å)*	49.05 – 2.01 (2.02 – 2.01)	49.02 – 2.10 (2.10 - 2.23)	48.32 – 1.91 (1.91 – 2.03)
Total no. of reflections	502370 (60962)	651606 (75997)	682594 (37102)
No. of unique reflections	145908 (22740)	66656 (10239)	147921 (16579)
R_{meas} (%)	16.2 (217.6)	14.2 (426.6)	14.5 (227.7)
I / σ	6.32 (0.45)	11.49 (0.39)	7.39 (0.35)
Completeness (%)	99.0 (95.1)	99.2 (95.7)	93.3 (64.8)
Redundancy	3.44 (2.68)	9.8 (7.4)	4.61 (2.23)
$CC_{1/2}$ (%)	99.5 (17.8)	99.9 (15.7)	99.6 (18.5)
ISa	30.76	30.07	27.55
**Refinement**			
Resolution (Å)	46.28-2.01	46.23 – 2.10	44.45-1.92
No. of reflections	144284	126172	144954
R_{work}/ R_{free}	20.01 / 23.62	21.06 / 24.90	18.29 / 23.37
Coordinate error	0.34	0.48	0.31
**No. of atoms**			
Protein	12531	6126	12524
DNA (p/t/dNTP)	373/670/42	239/409/30	373/666/-
Water	277	88	382
**Average b-factors (Å)**			
Protein	55.52	73.23	49.92
DNA (p/t/dNTP)	58.78/60.46/35.48	75.12/75.21/49.02	59.41/60.78/-
Water	47.86	57.31	44.52
**R.m.s. deviations**			
Bond lengths (Å)	0.002	0.008	0.012
Bond angles (°)	0.508	0.977	1.214
**Ramachandran (%)**			
Favored/ Allowed/ Outlier	97.48 / 2.52 / 0.00	96.24 / 3.62 / 0.13	97.89 / 2.11 / 0.00

* Values in parentheses correspond to those in the outer resolution shell.

Table 1: Summary of data collection and refinement statistics for KOD-21nt, KOD-U and KOD-H.

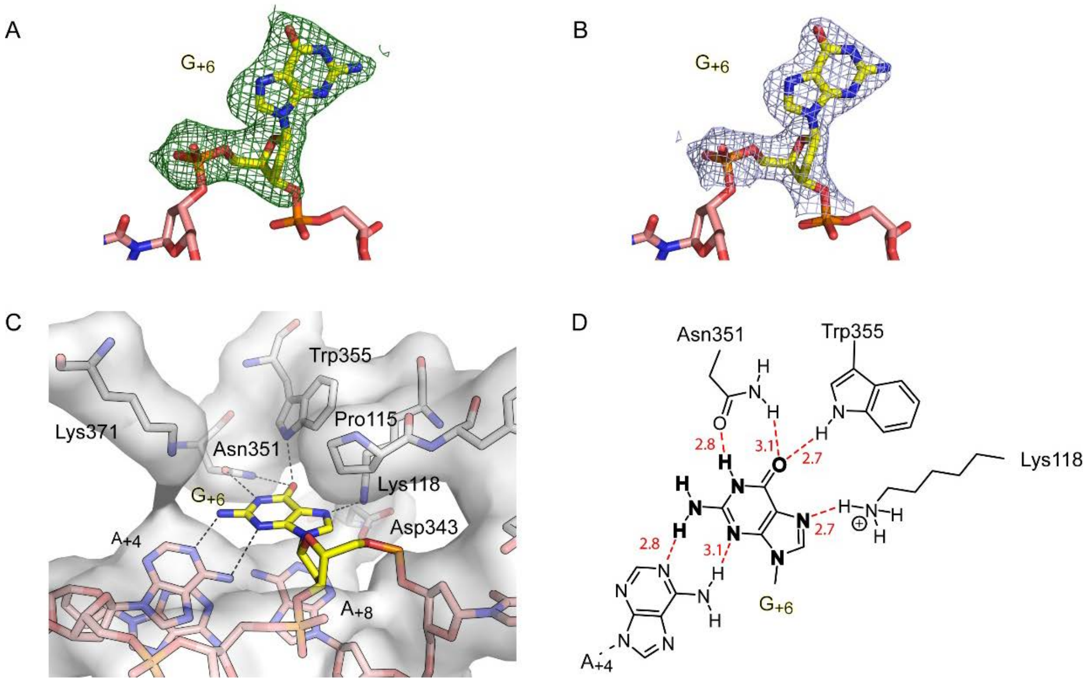

Figure S1. A) Polder map[1] for G_{+6} contoured at 3σ is shown in green. B) Final refined 2mFo-DFc map for G_{+6} contoured at 1σ is shown in blue. C) Binding position of template residue G_{+6} (yellow); residues surrounding G_{+6} in a radius of 5 Å are shown as sticks (protein residues: grey; template residues: salmon) and surface. Hydrogen bonding interactions are shown as dashed lines. D) Scheme of hydrogen bonding interactions of G_{+6} with distances given in Å.

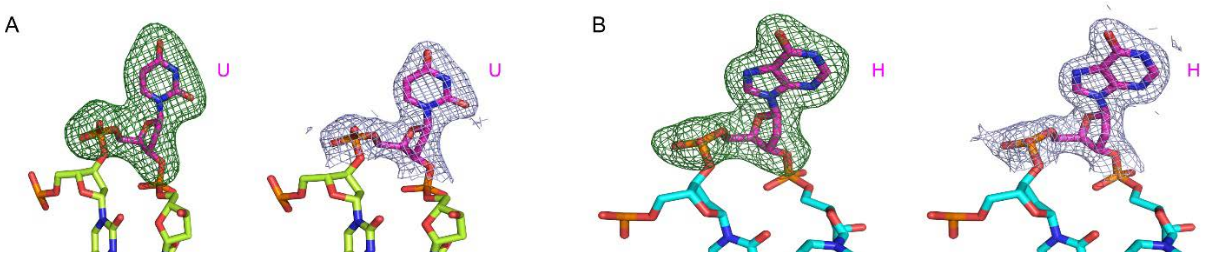

Figure S2. A) Polder map[1] contoured at 3σ is shown in green and final refined 2mFo-DFc map contoured at 1σ is shown in blue for the uracil residue (pink) at the template position +6 B) Same as in A for the hypoxanthine residue (pink).

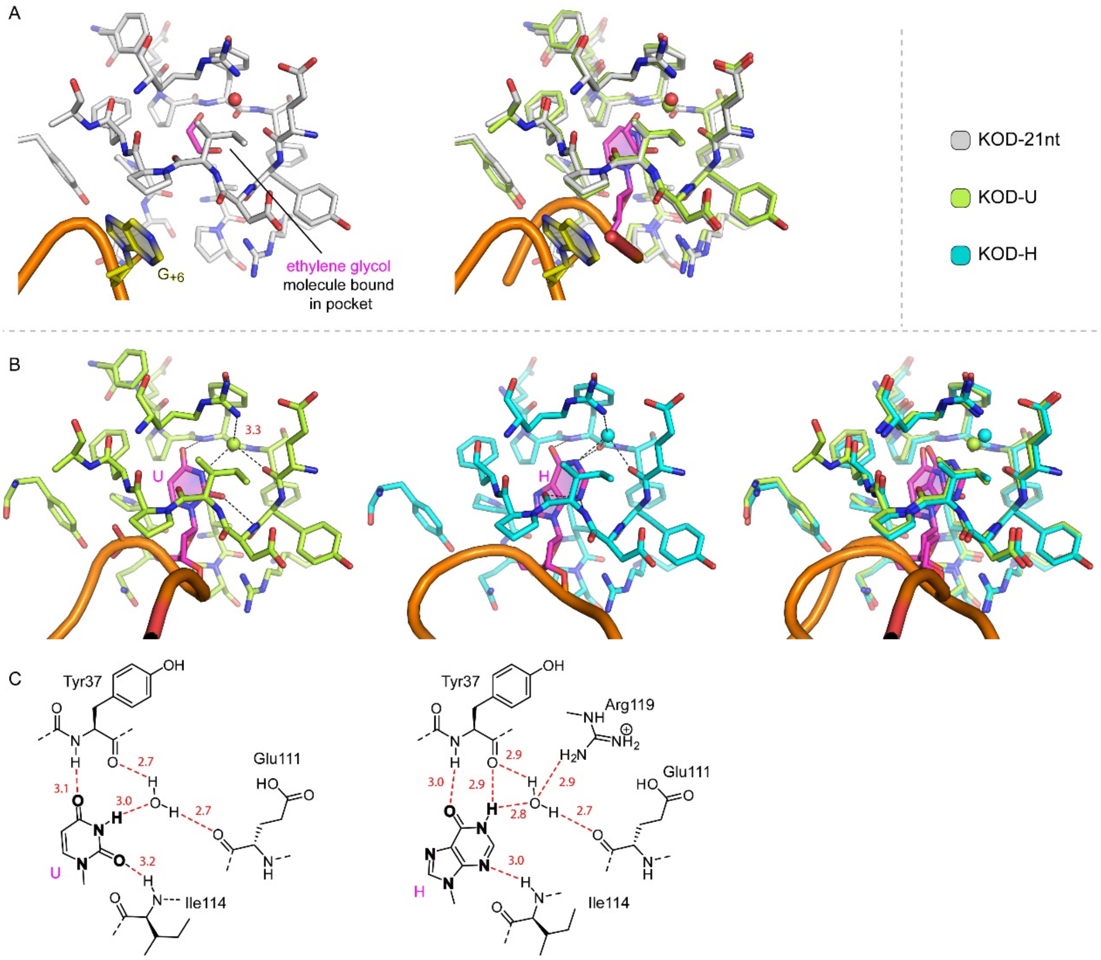

Figure S3. A) The ethylene glycol molecule bound in the uracil/hypoxanthine binding pocket is shown in pink. The binding pocket was defined by the protein residues surrounding U or H in a radius of 5 Å and includes the residues 7, 35-37, 90-91, 93-94, 97,111-117 and 119. An overlay of the binding pocket in KOD-21nt and KOD-U is shown on the right (superimposition was done based on the displayed protein residues including U or G_{+6}, respectively) and displays the similarity of the pocket. B) Comparison of the uracil/hypoxanthine binding pocket in KOD-U and KOD-H. The pockets are shown separately and as an overlay on the right (superimposition was done as in A). C) Interaction patterns of uracil and hypoxanthine in the binding pocket. Hydrogen bonds up to 3.2 Å distance are indicated and distances are given in Å. In the interaction pattern of KOD-U Arg119 is not shown as the distance of the hydrogen bond is 3.3 Å as indicated in Figure S3 B.

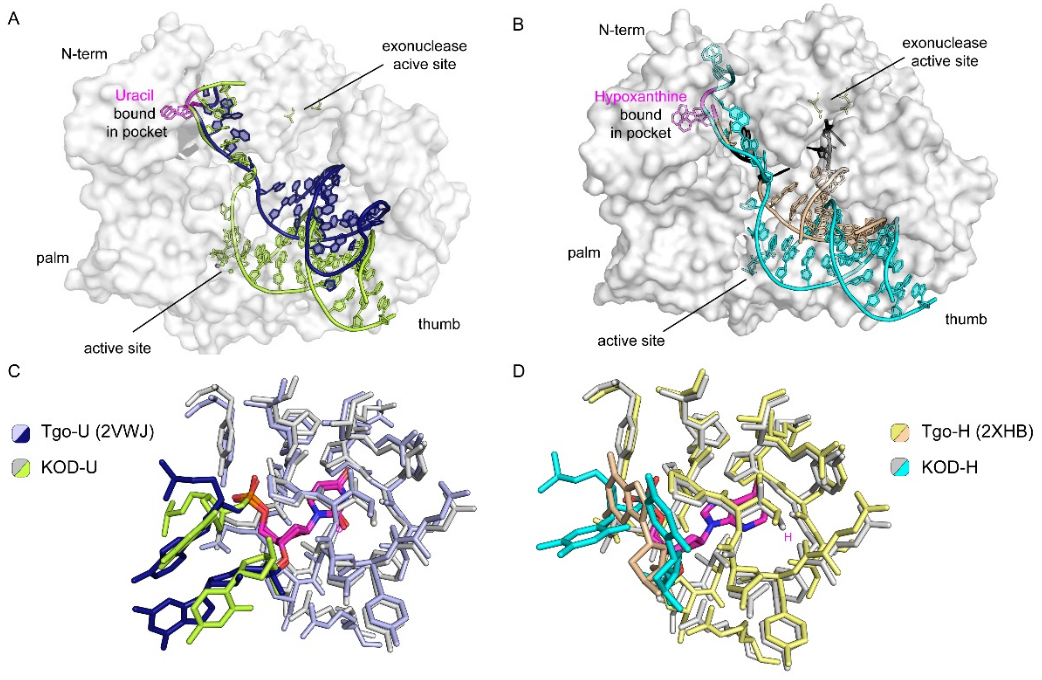

Figure S4. A, B) Different arrangement of the p/t complexes in the KOD and Tgo DNA Polymerase structures. A) Overlay of the overall structures KOD-U (green) and Tgo-U (dark blue). The p/t complexes are shown as cartoon and the surface of Tgo-U is shown in white. uracil residues are shown in pink. The polymerase and exonuclease active sites are indicated. B) Overlay of the overall structures KOD-H (cyan) and Tgo-H (sand). The p/t complexes are shown as cartoon and the surface of Tgo-H is shown in white. hypoxanthine residues are shown in pink. The complementary but dissociated nucleotides of the primer and template in Tgo-H are shown in black. C, D) Residues of the uracil/hypoxanthine binding pocket superpose well in KOD and Tgo DNA Polymerases. C) Superimposition of KOD-U and Tgo-U based on the uracil residue. All residues surrounding the uracil are shown as sticks. D) Superimposition of KOD-H and Tgo-H based on the hypoxanthine residues. All residues surrounding the hypoxanthine are shown as sticks.

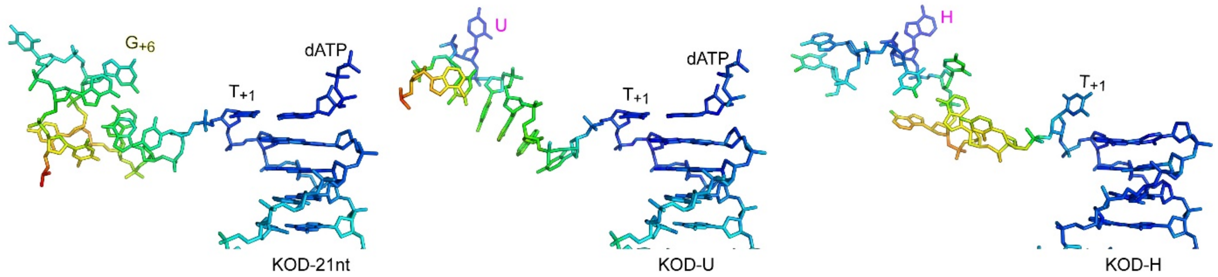

Figure S5. B-factors of the p/t duplexes in KOD-21nt, KOD-U and KOD-H. Flexibility color code goes from low flexibility (dark blue) to high flexibility (red).

[1] D. Liebschner, P. V. Afonine, N. W. Moriarty, B. K. Poon, O. V. Sobolev, T. C. Terwilliger and P. D. Adams, *Acta Cryst. D*, **2017** *73*, 148-157
